# Molecular Characterization of *Echinococcus granulosus* Sensu Lato from Farm Animals in Egypt

**DOI:** 10.1371/journal.pone.0118509

**Published:** 2015-03-11

**Authors:** Said Amer, Ibrahim B. Helal, Evelyne Kamau, Yaoyu Feng, Lihua Xiao

**Affiliations:** 1 Division of Foodborne, Waterborne and Environmental Diseases, National Center for Emerging and Zoonotic Infectious Diseases, Centers for Disease Control and Prevention, Atlanta, Georgia, United States of America; 2 Department of Zoology, Faculty of Science, Kafr El sheikh University, Kafr El Sheikh, Egypt; 3 Department of Zoology, Faculty of Science, Tanta University, Tanta, Egypt; 4 Institute of Systems and Synthetic Biology, ISSB, Universite d’Evry val d’Essonne, France; 5 State Key Laboratory of Bioreactor Engineering, School of Resources and Environmental Engineering, East China University of Science and Technology, Shanghai, People’s Republic of China; Aga Khan University Hospital Nairobi, KENYA

## Abstract

Little is known on the diversity and public health significance of *Echinococcus* species in livestock in Egypt. In this study, 37 individual hydatid cysts were collected from dromedary camels (n=28), sheep (n=7) and buffalos (n=2). DNA was extracted from protoscoleces/germinal layer of individual cysts and amplified by PCR targeting nuclear (actin II) and mitochondrial (COX1 and NAD1) genes. Direct sequencing of amplicons indicated the presence of *Echinococcus canadenesis* (G6 genotype) in 26 of 28 camel cysts, 3 of 7 sheep cysts and the 2 buffalo derived cysts. In contrast, *Echinococcus granulosus* sensu stricto (G1 genotype) was detected in one cyst from a camel and 4 of 7 cysts from sheep, whereas *Echinococcus ortleppi* (G5 genotype) was detected in one cyst from a camel. This is the first identification of *E*. *ortleppi* in Egypt.

## Introduction

Cystic echinococcosis (CE) is a cosmopolitan zoonotic disease caused by infection with the cestode *Echinococcus granulosus* sensu lato (s.l.). The domestic life cycle involves the ingestion of parasite eggs, derived from the final host (dogs and other canids), by an intermediate host belonging to a wide range of mammalian species including humans [1,2]. Recently, the World Health Organization (WHO) has included echinococcosis as part of a Neglected Zoonosis subgroup in its 2008–2015 strategic plans for the control of neglected tropical diseases [3]. The disease contributes to the poor overall development and work productivity in the endemic areas. CE remains highly endemic in pastoral communities, particularly in regions of South America, the Mediterranean littoral, Eastern Europe, East Africa, the Near and Middle East, Central Asia, China and Russia, with several millions of humans infected [4,5]. It is responsible for approximately 1% admissions to surgical wards in some countries such as Iran [6]. It is estimated that CE results in annual economic losses of several billion dollars in livestock sector due to low performance, morbidity and/or mortality of infected animals, and condemnation of infected organs of slaughtered animals [7].

Based on the biological and molecular studies, *E*. *granulosus* s.l. comprises a number of forms that differ substantially in infectivity, host range and genetic characteristics [2]. At least 10 strains (G1–10) of *E*. *granulosus* s.l. have been described [8], forming 4 major clades (G1–3, G4, G5, G6–G10) [9,10]. Recent re-evaluations of *Echinococcus* species strongly suggests that the genotypes G1 to G5 should be reclassified as *E*. *granulosus* sensu stricto (s.s.; G1 to G3), *E*. *equinus* (G4), and *E*. *ortleppi* (G5) [11]. There is also strong support for species status of genotypes G6 to G10 (*E*. *canadensis*) and the lion strain (*E*. *felidis*). This extensive biologic variation in *E*. *granulosus* may influence lifecycle patterns, pathology, antigenicity, transmission dynamics, and sensitivity to chemotherapeutic agents. For example, *E*. *canadensis* G6–G7, *E*. *equinus*, *E*. *granulosus* s.s., and *E*. *ortleppi* are transmitted mainly through domestic cycles [12]. The diagnosis of *Echinococcus* species involved might therefore have implications for the design and development of prevention and control measures, diagnostic assays, and drugs [13,14].

In Middle East and Africa, CE is a significant public health problem with high endemicity in the Arabic North Africa [15]. A considerable body of molecular data on *E*. *granulosus* s.l. from various intermediate and definitive hosts in African countries has indicated the prevalence of G1 strain (*E*. *granulosus* s.s.) and G6 strain (*E*. *canadensis*) in this area of the world [16–19]. Thus far, studies from Egypt are mostly phenotypic investigations [20,21], with few data available on genetic identity of the parasite. Among the few molecular studies, two [22,23] used RAPD banding patterns in characterizing hydatid cysts from several hosts and two [24,25] used PCR-sequence analysis of mitochondrial markers on cysts from a few hosts.

The present study was conducted to extend the knowledge on the identity of *E*. *granulosus* s.l. cysts collected from a range of livestock (camels, sheep, and buffalos) in Egypt. To this end, hydatid cysts were characterized by PCR-sequence analysis of the partial nuclear gene actin II and mitochondrial genes cytochrome C oxidase subunit 1 (COX1) and NADH dehydrogenase 1 (NAD1).

## Materials and Methods

### Ethics statement

The study was approved by the Institutional Committee of the Post-graduate Studies and Research at Kafr El Sheikh University, Egypt. Hydatid cysts were collected from slaughtered animals during post-mortem inspection by veterinary officers at the Al Basatein Abattoir, Cairo, Egypt, during April-October, 2011. Formal consent and permission for research use of hydatid cysts were obtained from both the university and abattoir veterinarians. No experiment was conducted on live animals.

### Cyst collection

A total of 37 hydatid cysts were collected from dromedary camels (*Camelus dromedarius*; n = 28), sheep (*Ovis aries*; n = 7) and buffalos (*Bubalus bubalis*; n = 2). All camel derived cysts except for 2 (from the liver) were collected from the lung, whereas all sheep and buffalo derived cysts were collected from the liver. To confirm the potential genetic diversity, only one cyst was collected from each animal. Individual cysts were placed in sterile saline solution in numbered plastic cups and transported within 6 hours to the laboratory in ice box. To evaluate the cyst fertility, cyst contents were aseptically aspirated, transferred into sterile Petri dishes, and examined for the presence of protoscoleces (fertile cysts). Protoscoleces were collected from individual fertile cysts, whereas germinal layer was collected from individual infertile cysts under aseptic conditions. Collected materials were washed three times with sterile saline solution and fixed in 95% ethanol.

### DNA extraction and PCR amplification

Materials from individual cysts were washed off ethanol with distilled water by centrifugation. DNA extraction was performed using the QIAamp DNA Mini Kit (Qiagen, Maryland, USA), following manufacturer-recommended procedures. A 266-bp fragment of the nuclear gene actin II was amplified using the primers described by Da Silva et al. [26], and 396- and 488-bp fragments of mitochondrial genes COX1 and NAD1, respectively, were amplified using the primers by Bart et al. [27]. PCR was done in 50 μl reaction mixture consisted of 1 × GeneAmp PCR buffer (Applied Biosystems, Foster City, CA), 200 μM of dNTP (Promega, Madison, WI), 3 mM MgCl_2_, 260 nM primers and 1.5 units of *GoTaq* DNA polymerase (Promaga). PCR cycles consisted of denaturation at 95°C for 5 min; 35 cycles of denaturation at 94°C for 30 s, annealing at 56°C (for NAD1) or 60°C (for actin II and COX1) for 30 sec and extension at 72°C for 50 s; and a final extension step at 72°C for 10 min. PCR products were analyzed by 1.5% agarose gel electrophoresis.

### DNA sequence analysis

PCR products were sequenced directly using the Big Dye Terminator v3.1 Cycle Sequencing Kit (Applied Biosystems) on an ABI 3130 Genetic Analyzer (Applied Biosystems). Sequences were assembled using the ChromasPro (version 1.5) software (http://technelysium.com.au/?page_id=27). The accuracy of data was confirmed by bi-directional sequencing. The obtained sequences were aligned with each other and reference sequences using ClustalX (http://www.clustal.org/) to determine the genotype of *Echinococcus* isolates. Unique nucleotide sequences generated in this study were deposited in GenBank under accession numbers AB921019 to AB921053 for actin II sequences, AB921054 to AB921090 for COX1 sequences, and AB921091 to AB921125 for NAD1 sequences.

### Phylogenetic analysis

In the present study, the haplotype approach described by Abushhewa et al. [16] was adopted to infer phylogeny, using the Bayesian analysis implemented in MrBayes software (version 3.2.2) (http://mrbayes.sourceforge.net/) and the Metropolis Coupled Markov Chain Monte Carlo (MCMCMC) method to estimate posterior distribution of parameters. Haplotype segregation in the generated sequences was determined using the TCS (version 1.21) software (http://darwin.uvigo.es/software/tcs.html). Reference sequences were compiled from previous studies (Table 1), with *Taenia saginata* as an outgroup. Nexus files of aligned COX1 and NAD1 gene fragment sequences were created using ClustalX [29], while aligned matrices of COX1 and NAD1 were concatenated using Mesquite software (http://mesquiteproject.org) In MrBayes, the model of sequence evolution was specified with two substitution types (*nst = 2*), transitions and transversions, for analysis of the input data matrix. The 50% majority rule consensus tree generated was viewed and printed using the Mesquite program.

**Table 1:** GenBank accession numbers of COX1 and NAD1 of *Echinococcus* species/ genotypes/haplotype used in phylogenetic analysis in the present study.

Parasite species/genotype/haplotype	Host origin	Locality	Accession No. for COX1	Accession No. for NAD1
Haplotype 1 (G1)*	Camel	Egypt	AB921054	AB921091
Haplotype 2 (G1)*	Sheep	Egypt	AB921090	AB921125
Haplotype 3 (G5)*	Camel	Egypt	AB921055	AB921092
Haplotype 4 (G6)*	Camel	Egypt	AB921058	AB921095
Haplotype 5 (G6)*	Camel	Egypt	AB921068	AB921105
Haplotype 6 (G6)*	Camel	Egypt	AB921075	AB921111
Haplotype 7 (G6)*	Camel	Egypt	AB921083	AB921119
Haplotype 8 (G6)*	Camel	Egypt	AB921084	AB921120
Haplotype 9 (G6)*	Camel	Egypt	AB921085	AB921121
G1–3	Cattle	Libya	HM636639	HM636643
G1–3 (G1)	Cattle	Libya	HM636641	HM636644
G1–3 (G1)	Cattle, camel	Iran	FJ796205	FJ796208
G1–3 (G1)	Human, Cattle	Turkey	HQ717150	HQ717151
G1–3 (G1)	Human	Turkey	HQ717148	HQ717153
G1	Human	Iran	KF612390	KF612360
G1	Human	Iran	KF612376	KF612349
G1	Sheep	Greece	DQ856467	DQ856470
G1	Sheep	United Kingdom	AF297617	AF297617
G2	Sheep	Australia	M84662	AJ237633
G1–3 (G3)	Camel	Iran	FJ796206	FJ796214
G3	Buffalo	India	M84663	AJ237634
G3	Human	Iran	KF612397	KF612369
G3	Sheep	Greece	DQ856466	DQ856469
G4	Horse	India	M84664	AJ237635
G5	Cattle	Netherlands	M84665	AJ237636
G6–10 (G6)	Camel	Iran	FJ796207	FJ796216
G6	Camel	Africa	M84666	AJ237637
G6	Cattle, camel	Libya	HM636638	HM636642
G6	Human	Iran	KF612400	KF612372
G6	Camel	Kazakhstan	NC_011121	NC_011121
G6–10 (G7)	Human	Turkey	HQ717155	HQ717154
G7	Goat	Greece	DQ856468	DQ856471
G7	Pig	Poland	M84667	AJ237638
G8	Moose	USA	AB235848	AJ237643
G10	Reindeer	Finland	AF525457	AF525297
*E. shiquicus*	Pika	China	AB208064	AB208064
*E. felidis*	Lion	Uganda	EF558356	EF558357
*E. oligarthrus*	Rodent	Panama	M84671	AJ237642
*E. vogeli*	Rodent	South America	M84670	AJ237641
*E. multilocularis*	Human	Alaska, China	M84668	AJ237639
*E. multilocularis*	Rodent	Germany	M84669	AJ237640
*Taenia saginata*	Human	Belgium	NC_009938	NC_009938

*Sequences generated in this study.

## Results

### Distribution of *Echinococcus* genotypes

Examination of the collected cysts indicated that 3 of 28 cysts derived from dromedary camels were infertile; all others were fertile cysts with protoscoleces. Successful PCR amplification and DNA sequencing were achieved for DNA from all fertile and infertile cysts at all three genetic loci. Genotype identifications at all three genetic loci were in complete agreement for all isolates. Blast search of the obtained sequences derived from camel isolates indicated the occurrence of G6 genotype (*E*. *canadenesis*) in 26 of 28 isolates, and G1 (*E*. *granulosus* s.s.) and G5 (*E*. *ortleppi*) genotypes in one isolate each (35406 and 35408, respectively). Among sheep isolates, G6 and G1 genotypes were seen in 3 and 4 of the 7 isolates, respectively. The 2 isolates derived from buffalo both belonged to the G6 genotype.

### Sequence polymorphism in actin II

The alignment of actin II sequences indicated no intra-genotype sequence variation within G6 or G1 genotypes. Sequences of the G6 genotype showed complete identity (100%) to sequences AF528500 generated from hydatid cysts from camels in Algeria [28], and DQ341548 and DQ341551 from cattle and humans from Mauritania [29]. Sequences generated from G1 isolates showed complete identity to relevant sequences in GenBank database from various geographic areas and hosts (FJ997234, FJ997233, EF179175, EF125692, AF528499 and AF528498). The G5 sequence generated from isolate 35408 had one nucleotide substitution (T to C) at position 208 of the reference sequence AF003748 from cattle, and 4 and 7 substitution comparing to sequences AF003749 and AF528500 derived from pigs and camels [26,28], respectively.

### Sequence polymorphism in COX1

The alignment of COX1 sequences showed all except one from the G6 genotype in this study were identical. These sequence showed 100% identity to published sequences of G6 derived from different hosts such as AB208063 from camels in Kazakhstan [9], AB458677 from goats in Peru [30], and AB688142 from humans in Russia (direct submission). Only one sequence (35420) had a single nucleotide substitution of C to T at position of 327 of the generated sequences, but was identical to sequence AB650535 generated from camels in Ethiopia [31]. The sequence of G5 genotype showed 99% identity with a single nucleotide substitution of C to T comparing to reference sequences (FJ744757, AB235846 and JX854035) at position 52. No intra-sequence variations were noticed in sequences of the G1 genotype; they were identical to other sequences (KC660075, AB786664, JQ317997, FN646371 and HM598452) in GenBank.

### Sequence polymorphism in NAD1

The alignment of NAD1 sequences of G6 genotype isolates showed the presence of 4 types of sequences. The first one contained the majority of sequences and had a complete identity to sequences HM749616, HM749616, HM749617 and HM749618 from camels in Iran [32] and JN637176 from a camel in Sudan [33]. The second type had 4 sequences (37535, 37544, 37545 and 37546) and 2 nucleotide substitutions (both T to A) at positions 315 and 321 comparing to the first type. The third type had only one sequence (37543) and 3 nucleotide substitutions (all T to A) at positions 313, 315 and 321. The fourth type had one sequence (37541) and a single nucleotide substitution (G to C) at position 386. Likewise, two types of G1 sequences were obtained in the study. The first type had 4 sequences and 100% identity to reference sequences AB786664, JQ356721, JF946624, JF946623, GQ358010 HM055622 and others from different countries and hosts. The other sequence was from isolate 37551 and had 2 nucleotide substitutions at positions 281 (T to C) and 321 (T to A). In contrast, isolate 35408 of the G5 genotype generated a sequence identical to G5 sequences JN637177 from camel cysts in Sudan and AB235846 from cattle cysts in Argentina [9], while showed 99% identity to sequence AJ237636 from cattle cysts in the Netherlands [34].

### Phylogeny of *Echinococcus* genotypes

**Fig 1:**
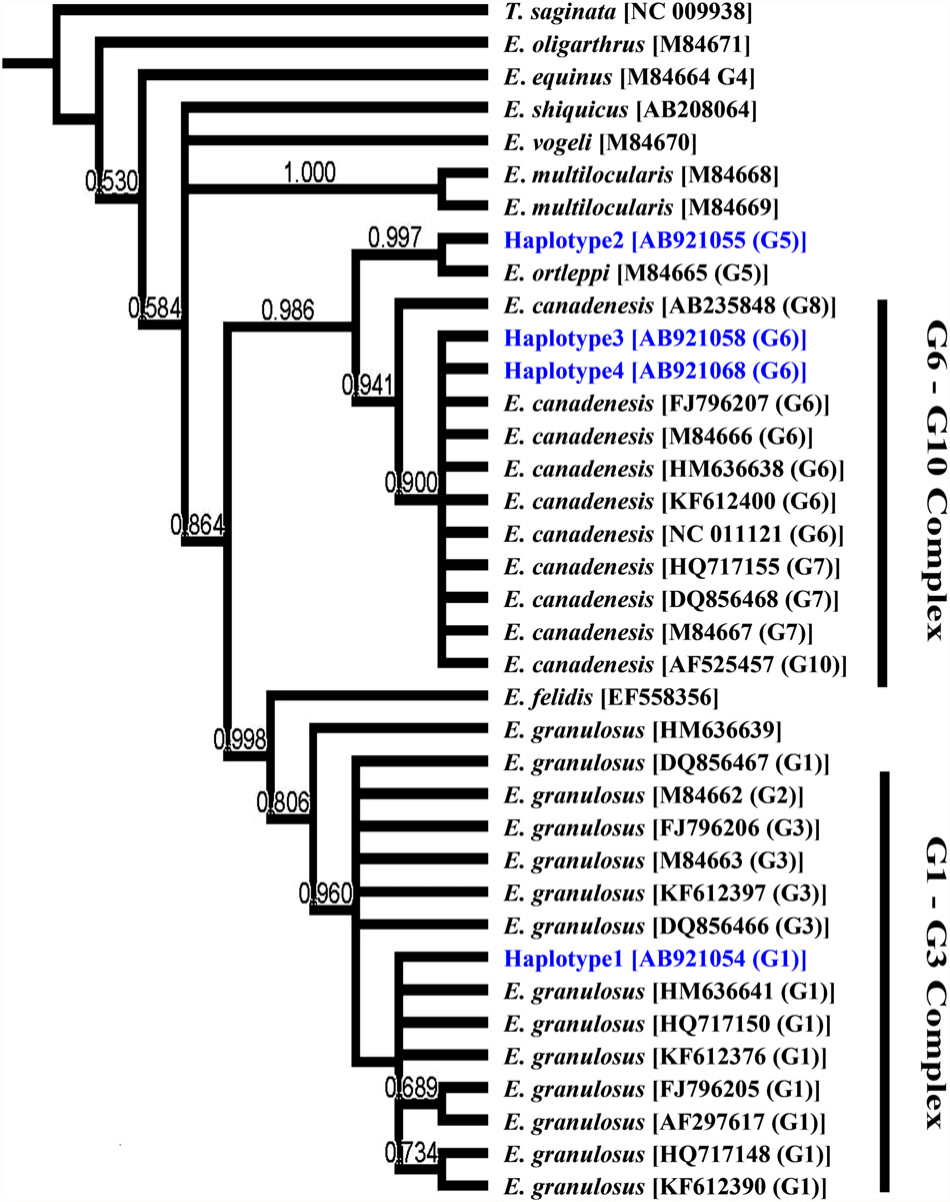
Phylogenetic relationships of *Echinococcus granulosus* sensu lato isolates from Egypt compared to reference sequences of different *Echinococcus* species in database. Evolutionary relationship was inferred based on COX1 sequences (Table 1) using the Bayesian inference (BI) implemented in MrBayes software (version 3.2.2). *Taenia saginata* (NC_009938) was used as the outgroup.

**Fig 2:**
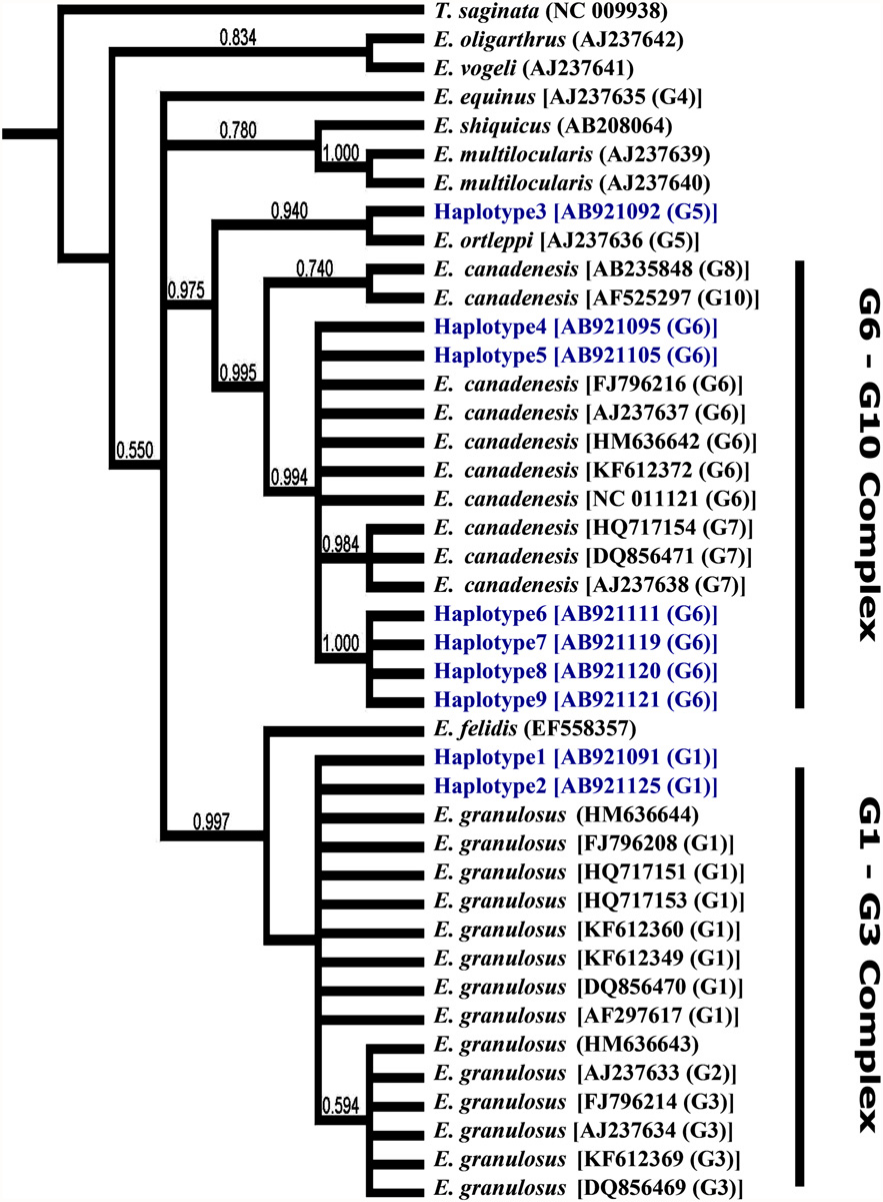
Phylogenetic relationships of *Echinococcus granulosus* sensu lato isolates from Egypt compared to reference sequences of different *Echinococcus* species in database. Evolutionary relationship was inferred based on NAD1 sequences (Table 1) using the Bayesian inference implemented in MrBayes software (version 3.2.2). *Taenia saginata* (NC_009938) was used as the outgroup.

**Fig 3:**
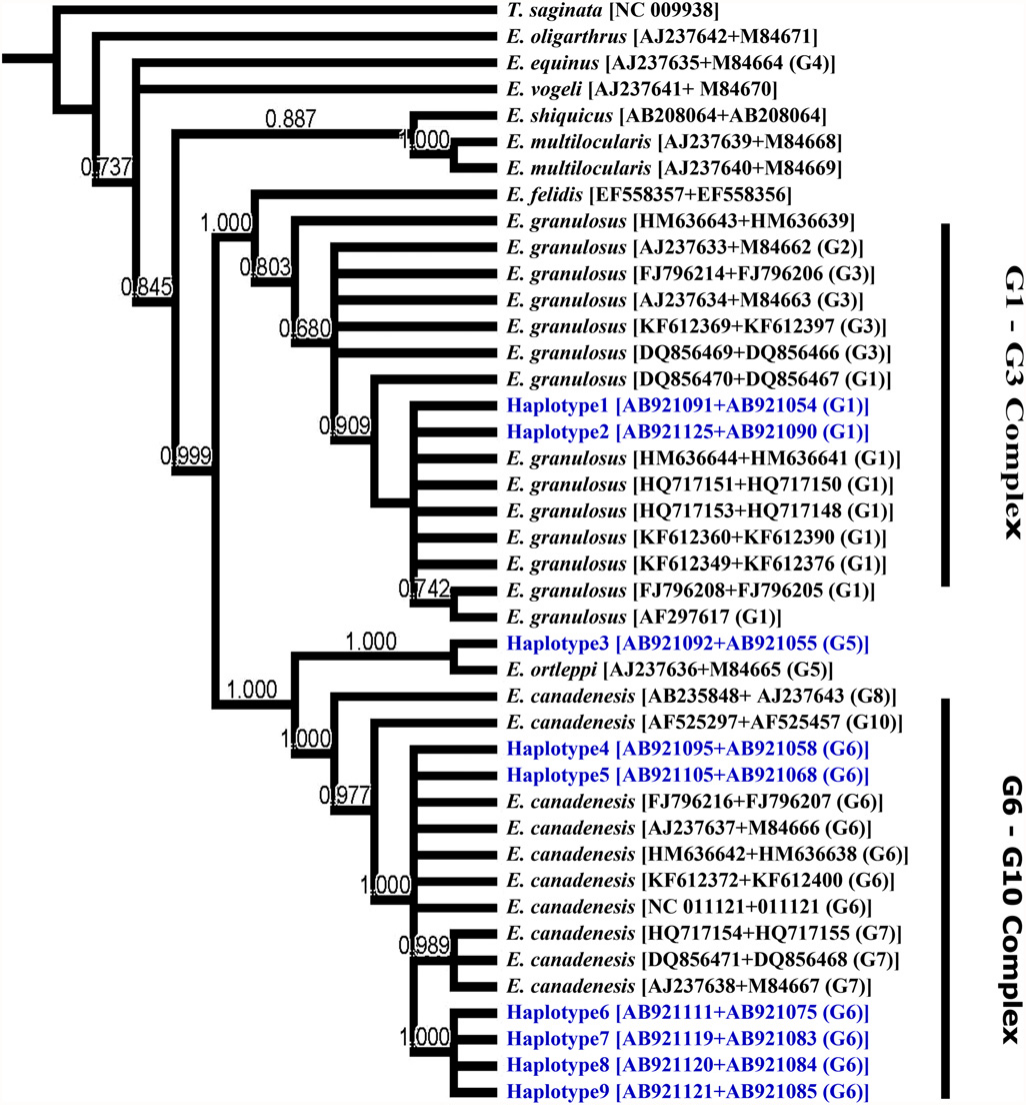
Phylogenetic relationships of *Echinococcus granulosus* sensu lato isolates from Egypt compared to reference sequences of different *Echinococcus* species in database. Evolutionary relationship was inferred based on concatenated COX1 and NAD1 sequences (Table 1) using the Bayesian inference implemented in MrBayes software (version 3.2.2). *Taenia saginata* (NC_009938) was used as the outgroup.

Altogether, 4 haplotypes were detected in COX1 sequences with 2 haplotypes within the G6 genotype, whereas 9 haplotypes were identified at the NAD1 locus with 2 haplotypes within the G1 genotype and 6 haplotypes within the G6 genotype. Phylogenetic trees constructed based on the COX1 (Fig. 1) and NAD1 (Fig. 2) sequences were similar to a tree (Fig. 3) constructed based on the concatenated sequences of both genes. In particular, haplotype 1 of COX sequences grouped with reference sequences from the G1–3 complex, particularly the G1 genotype, with strong bootstrap support (0.90). Haplotype 2 clustered with the *E*. *ortleppi* (G5) group, separating from the G6–10 complex with high bootstrap support (0.98). Haplotypes 3 and 4 grouped with the G6 genotype in the G6–G10 complex (bootstrap value = 0.9). Similar distributions of haplotypes within G1–3 and G6–10 complexes were seen in trees constructed based on NAD1 and concatenated sequences. At the genotype level, G5 genotype (*E*. *ortleppi*) formed a monophyletic group with G6–10 complex. Similarly, *E*. *felidis* was more related to *E*. *granulosus* s.s. (G1–G3) forming a sister phylogenetic group (99.0–1.00). Other species were more distant. Thus, *E*. *oligarthrus* was very genetically different from other recognized *Echinococcus* species and genotypes (Figs. 1–3). Likewise, *E*. *vogeli*, *E*. *equines* (G4), *E*. *multilocularis* and *E*. *shiquicus* (0.737) were distinct from most *E*. *granulosus* s.l. genotypes (G1–G3, G5, and G6–10). Interestingly, *E*. *vogeli* grouped with *E*. *oligarthrus* in the tree inferred using NAD1 sequences (Fig. 2).

## Discussion

CE is endemic in both animals and humans in Egypt [24]. Domestic intermediate hosts are major reservoirs for the disease in humans. Accumulated reports indicated that various livestock are susceptible to hydatid infection in Egypt, with particularly high incidence in camels and sheep [20,35]. In addition, *Echinococcus* infection is common in stray dogs in Egypt [36], who are usually roaming on streets and near slaughterhouses, feeding on offal of slaughtered animals or carcasses of dead animals in rural areas. Stray dogs also have free access to yards and fields of domestic animals, contaminating the environment with *Echinococcus* eggs. It is reported that CE is typically a disease of pastoral communities, but less common in agricultural communities [2,18]. Even though Egypt has few pastoral communities, the abundance of stray dogs and common home/street slaughter practices, especially during the festival events, facilitate the establishment of dog-livestock transmission cycle of *Echinococcus* spp. and increase the risk of human infection [37].

Results reported here indicated that three *Echinococcus* species are present in farm animals in Egypt, including *E*. *granulosus* (sheep genotype or G1), *E*. *canadensis* (camel genotype or G6), and *E*. *ortleppi* (cattle genotype or G5). The dominance (83.8%) of G6 over the other genotypes (13.5% for G1 and 2.7% for G5) in this study is in agreement with recent reports from Egypt [24,25], which showed exclusive occurrence of G6 in camels and pigs and *E*. *equinus* (G4 genotype) in donkeys, and a predominance of G6 with a small number of G1 infections in humans. In contrast, Abd El Baki et al. [38] claimed that G1 was common in humans, camels and sheep using strain specific PCR. Results of the present study have shown a higher diversity of *E*. *granulosus* s.l. in Egypt than previously reported, with *E*. *ortleppi* being reported for the first time.

The distribution of *Echinococcus* genotypes differs from country to country and from hosts to hosts. Circulation of the G6 genotype in humans, dromedary camels and cattle was reported in Mauritania [29,39]. This also appears to be the case in Sudan, where G6 is the dominant genotype in humans, camels, cattle, sheep and goats, with some G5 infections in cattle and camels [18,19,33]. Elsewhere, substantial percentages of G6 genotype have been reported in human cases in Argentina (~37%) [40] and Kenya (17%) [17]. Interestingly in Libya, G1 is the exclusive genotype in humans with some occurrence in cattle, whereas G6 dominates in cattle and camels [16]. G1 is also the common genotype in different hosts in Ethiopia [31], Tunisia [39], Palestine [41], Iran [42], India [43], China [44] and Mongolia [45]. *Echinococcus granulosus* s.s. (G1- G3 complex) is also the major genotype in humans, cattle, sheep and goats in many European and Latin American countries [27,46,47].

The three *Echinococcus* genotypes detected in this study, G1, G5 and G6, are all known human pathogens [48,49], imposing a significant public health concern. Strains variation may play an important role in not only transmission patterns but also pathogenicity, fertility, and growth rate of hydatid cysts [48]. Considering the fact that most camels for human consumption in Egypt are imported from Sudan, imported camels could be a source of *E*. *canadensis* in Egypt. This issue cannot be addressed in the present study because of the low number of cysts analyzed and low resolution of typing tools used. A larger study using a number of isolates from diverse hosts, including humans and stray dogs, from multiple geographic areas is needed to better understand the epidemiology of CE in Egypt.
